# Lymphotoxin β Receptor Controls T Cell Progenitor Entry to the Thymus

**DOI:** 10.4049/jimmunol.1601189

**Published:** 2016-08-22

**Authors:** Beth Lucas, Kieran D. James, Emilie J. Cosway, Sonia M. Parnell, Alexi V. Tumanov, Carl F. Ware, William E. Jenkinson, Graham Anderson

**Affiliations:** * Medical Research Council Centre for Immune Regulation, Institute for Immunology and Immunotherapy, University of Birmingham, Birmingham B15 2TT, United Kingdom;; † Trudeau Institute, Saranac Lake, NY 12983; and; ‡ Infectious and Inflammatory Diseases Research Center, Sanford Burnham Medical Research Institute, La Jolla, CA 92037

## Abstract

The recruitment of lymphoid progenitors to the thymus is essential to sustain T cell production throughout life. Importantly, it also limits T lineage regeneration following bone marrow transplantation, and so contributes to the secondary immunodeficiency that is caused by delayed immune reconstitution. Despite this significance, the mechanisms that control thymus colonization are poorly understood. In this study, we show that in both the steady-state and after bone marrow transplant, lymphotoxin β receptor (LTβR) controls entry of T cell progenitors to the thymus. We show that this requirement maps to thymic stroma, further underlining the key importance of this TNFR superfamily member in regulation of thymic microenvironments. Importantly, analysis of the requirement for LTβR in relationship to known regulators of thymus seeding suggests that it acts independently of its regulation of thymus-homing chemokines. Rather, we show that LTβR differentially regulates intrathymic expression of adhesion molecules known to play a role in T cell progenitor entry to the thymus. Finally, Ab-mediated in vivo LTβR stimulation following bone marrow transplant enhances initial thymus recovery and boosts donor-derived T cell numbers, which correlates with increased adhesion molecule expression by thymic stroma. Collectively, we reveal a novel link between LTβR and thymic stromal cells in thymus colonization, and highlight its potential as an immunotherapeutic target to boost T cell reconstitution after transplantation.

## Introduction

In the thymus, immature lymphoid progenitors undergo a complex differentiation program that biases thymocyte development toward the generation of self-tolerant MHC-restricted T cells (1). Importantly, the hemopoietic progenitors that colonize the thymus are generated in extrathymic sites, and so T cell development depends on thymic colonization by migrant progenitors (2, 3). As the thymus does not contain hemopoietic stem cells with long-term self-renewal capacity, there is an ongoing requirement for this recruitment process, and this is important for several reasons. First, it creates successive waves of thymopoiesis to maintain long-term T cell production (4, 5). Second, it establishes competition for intrathymic niches that limits the self-renewal of intrathymic progenitors (6–8). Importantly, absence of competition manifests as T cell acute lymphoblastic leukemia, indicating that thymus seeding is part of an intrathymic tumor suppression mechanism that requires constant replacement of the immature thymocyte pool (9).

Although lymphoid progenitors are known to enter the adult thymus via blood vessels at the corticomedullary junction (10), their rarity means that their exact nature remains unclear (11–13). However, insight into the mechanisms that control thymus colonization can be obtained by studying the frequency and requirements of CD4^−^CD8^−^CD44^+^CD25^−^CD117^+^ thymocytes that represent the earliest thymic progenitors (ETP) in the adult mouse thymus (13–16). Thus, thymus entry is recognized as a multistep process involving chemokines, adhesion molecules, and growth factors produced by thymic microenvironments. For example, thymic endothelial cells express VCAM-1, ICAM-1, and P-selectin (17–19) to enable the attachment of blood-borne lymphoid progenitors. Significantly, Ab blockade of VCAM-1/ICAM-1 impairs lymphoid progenitor entry to the thymus (20), whereas mice deficient in either P-selectin or its receptor PSGL-1 have fewer ETP and an increased availability of intrathymic niches (18). ETP express the chemokine receptors CXCR4, CCR7, and CCR9 (21–24), and the chemokines CCL19, CCL21, CCL25, and CXCL12 are all products of thymic stroma (21, 25, 26). Significantly, disruption of these molecules either individually or in combination results in impaired thymus seeding (22, 23, 27, 28). Importantly, however, although these studies emphasize the importance of the thymic microenvironment in the recruitment of lymphoid progenitors to the thymus, this process is still poorly understood and relatively few of its regulators are known.

The importance of thymus seeding is further emphasized by its regulation of immune system recovery that follows ablative therapy and bone marrow (BM) transplant (BMT), where limited thymus entry of donor progenitors slows down T cell reconstitution in comparison with other blood cell lineages (29, 30). Indeed, intrathymic progenitor niches are not saturated until at least 10 wk after BMT (29), suggesting that delayed T cell reconstitution is linked to inefficient thymus seeding. Interestingly, although PSGL-1 has been identified as an important regulator of thymus seeding after BMT (29), the cellular and molecular mechanisms that limit T cell recovery after transplant, and how they relate to the requirements of steady-state T cell development, remain poorly understood.

In this study, we show that mice lacking lymphotoxin β receptor (LTβR) demonstrate a dramatic reduction in the frequency of ETP, and that increased compensatory intrathymic progenitor proliferation accounts for their normal thymocyte numbers. Importantly, thymus transplant and BM chimera experiments show the requirement for LTβR maps to thymic stromal cells. We also show that LTβR differentially regulates thymic stromal expression of VCAM-1 and ICAM-1 but not P-selectin, which collectively represent adhesion molecules previously linked to thymus entry. Finally, we show that thymic recovery after BMT also requires LTβR, and that agonistic anti-LTβR treatment enhances donor-derived T cell reconstitution. Collectively, our findings identify a novel regulatory axis of T cell progenitor entry to the thymus, and they extend our understanding of the importance of LTβR in the functional control of thymic stromal microenvironments.

## Materials and Methods

### Mice

Adult wild-type (WT) C57BL/6 and congenic CD45.1^+^ C57BL/6 mice, and *Ltbr^−/−^* (31) and *plt/plt* (32) mice on a C57BL/6 background were used at 8–12 wk of age. All mice were housed at the Biomedical Services Unit at the University of Birmingham in accordance with local and national Home Office regulations.

### Abs and flow cytometry

For thymocyte and splenocyte analysis, tissues were enzymatically digested (33) using collagenase D (2.5 mg/ml; Roche) and DNase I (40 μg/ml; Roche). Cells were stained with Abs specific for CD44 (IM7), CD25 (PC61.5), CD117 (2B8), CD45 (30-F11), CD45.1 (A20), CD45.2 (104), CD4 (GK1.5), CD8β (53-6.7), TCRβ (597), and Foxp3 (FJK-16s), conjugated to Brilliant Violet (BV) 605, BV510, Pacific Blue, eFluor 450, PE, PE-Cy7, PerCP–eFluor 710, allophycocyanin–eFluor 780 and Alexa Fluor 700. Abs were purchased from eBioscience, BD Biosciences, or BioLegend. Foxp3 staining was performed using an intracellular Foxp3 kit purchased from eBioscience. Streptavidin-BV786 was used to reveal staining with biotinylated Abs. The following lineage markers were used: CD3ε (145-2C11), CD4 (GK1.5), CD8α (53-6.7), CD8β (H35-17.2), CD11b (M1/70), CD11c (N418), B220 (RA36B2), Ly-6G (RB6-865), NK1.1 (PK136), Ter-119 (TER-119), TCRβ (H57-597), and TCRδ (GL3). Prior to surface staining, cells were stained with a fixable viability dye (Near-IR stain; Invitrogen). For the analysis of stromal cells, thymuses were enzymatically digested (34) using collagenase dispase (2.5 mg/ml; Roche) and DNase 1 (40 μg/ml; Roche). Prior to surface staining, CD45^+^ cells were depleted using anti-CD45 microbeads and LD columns (Miltenyi Biotec) and then stained with a fixable viability dye (Near-IR stain; Invitrogen). Cells were stained with mAbs against CD45 (30-F11), EpCAM1 (G8.8), TER-119 (TER-119), podoplanin (8.1.1), CD31 (390), ICAM-1 (YN1/1/7/4), and VCAM-1 (429). Abs were conjugated to allophycocyanin, allophycocyanin–eFluor 780, PE, PE-Cy7, FITC, Alexa Fluor 700, BV605, or BV421. Data were acquired using a BD LSRFortessa and were analyzed using FlowJo software (Tree Star). Forward and side scatter gates were set to exclude nonviable and aggregated cells.

### BrdU incorporation

Mice were injected i.p. with 1.5 mg of BrdU (Sigma-Aldrich) and tissue was harvested 3 h later. Staining for BrdU was performed using the BrdU flow kit (BD Biosciences), according to the manufacturer’s instructions.

### Generation of BM chimeras

Recipient mice were lethally irradiated (2× 500 rad) and reconstituted i.v. with 5 × 10^6^ T cell–depleted adult BM preparations from CD45 congenically marked mice, as indicated. Depletion of T cells was performed using anti CD3-PE and anti-PE microbeads (Miltenyi Biotec) according to the manufacturer’s instructions. Mice were sacrificed at the indicated time points, and tissues were analyzed by flow cytometry. In some experiments, mice received 100 μg of agonistic anti-LTβR (35) or isotype control on days 1, 3, 5, 7, and 9, and in these experiments tissues were harvested for analysis at day 10 or day 28.

### Stromal cell isolation and PCR

Digested thymuses (34) were depleted of CD45^+^ cells using anti-CD45 microbeads (Miltenyi Biotec), in conjunction with LD columns. Cells were stained with Abs to CD45 (30-F11), EpCAM1 (G8.8), TER-119 (TER-119), and podoplanin (8.1.1), and CD45^−^EpCAM1^+^ thymic epithelial cells (TEC) and CD45^−^EpCAM1^−^podoplanin^+^ mesenchymes were FACS sorted using a MoFlo XDP (Beckman Coulter). CD31^+^ endothelial cells were sorted using anti-CD31 microbeads (Miltenyi Biotec) and MS columns, according to the manufacturer’s instructions. Sorted populations were analyzed by quantitative PCR (qPCR) for expression of the indicated genes exactly as described (36). Primer sequences are as follows: Actb (NM_007393) QuantiTect Mm_Actb_1_SG primer assay (Qiagen QT00095242); Ccl19 NM_(011888.2), forward, 5′-GCTAATGATGCGGAAGACTG-3′, reverse, 5′-ACTCACATCGACTCTCTAGG-3′; Ccl21a (NM_011124.4), forward, 5′-ATCCCGGCAATCCTGTTCTC-3′, reverse, 5′-GGGGCTTTGTTTCCCTGGG-3′; Ccl25 (NM_009138.3), forward, 5′-TTACCAGCACAGGATCAAATGG-3′, reverse, 5′-CGGAAGTAGAATCTCACAGCA-3′; Cxcl12 (NM_021704.3), forward, 5′-GCTCTGCATCAGTGACGGTA-3′, reverse, 5′-TGTCTGTTGTTGTTCTTCAGC-3′; Kitl (NM_013598.2), forward, 5′-CCCTGAAGACTCGGGCCTA-3′, reverse, 5′-CAATTACAAGCGAAATGAGAGCC-3′; Selp (NM011347.2), forward, 5′-CATCTGGTTCAGTGCTTTGATCT-3′, reverse, 5′-ACCCGTGAGTTATTCCATGAGT-3′.

### Thymus transplantation

Embryonic day 15 thymuses, organ cultured for 5 d in 1.35 mM 2-deoxyguanosine, were transplanted under the kidney capsule of WT mice and harvested after 6–8 wk (34).

### Statistical analysis

Data were analyzed using an unpaired *t* test. A *p* value <0.05 was considered significant. Statistical analysis was performed using GraphPad Prism. Data are represented as mean ± SEM.

## Results

### LTβR controls ETP frequency

**FIGURE 1.**
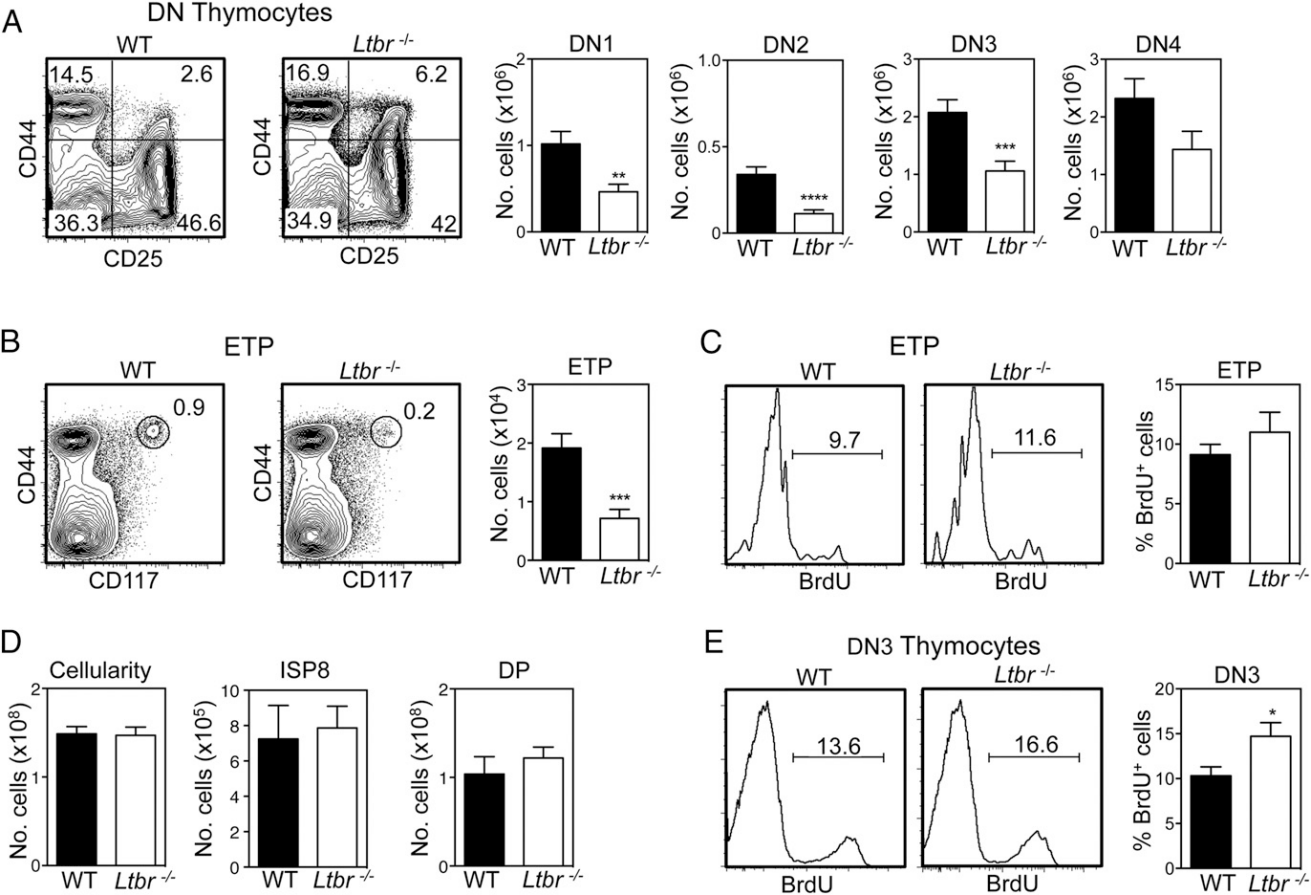
Reduced early thymus progenitors in LTβR^−/−^ mice. CD4^−^CD8^−^ (DN) thymocytes (**A**) and ETP (**B**) in WT and *Ltbr*^−/−^ thymus, gated on lineage^−^ and lineage^−^CD25^−^ cells, respectively. Representative FACS plots are shown (*n* ≥ 23). (**C**) Representative FACS plots and proportions of BrdU^+^ ETP in WT and *Ltbr*^−/−^ thymus (*n* = 10). (**D**) Total cellularity and numbers of immature single-positive 8 (ISP8) and CD4^+^CD8^+^ double-positive (DP) thymocytes in WT and *Ltbr*^−/−^ thymus (*n* ≥ 8). (**E**) BrdU incorporation and proportions of BrdU^+^ DN3 thymocytes from WT and *Ltbr*^−/−^ thymus (*n* ≥ 8). All data are from at least three independent experiments. *p < 0.05, **p < 0.01, ***p < 0.001, ****p < 0.0001.

Given the importance of the TNFR superfamily (TNFRSF) in the organization and development of functionally competent thymic stromal microenvironments, we screened a panel of mutant mice for evidence of impaired thymus colonization. Although no obvious alterations were found in *Tnfsf1^−/−^* and *Tnfrsf11b^−/−^* mice, we found that *Tnfrsf3^−/−^* mice (described here as *Ltbr^−/−^* mice) had reduced numbers of downstream early T cell progenitors, including those at the double-negative (DN) 1–3 stages of development (Fig. 1A). Importantly, we also saw a significant reduction in both the proportion and absolute number of lineage^−^CD44^+^CD25^−^CD117^+^ ETP in *Ltbr^−/−^* mice (Fig. 1B). Analysis of cellular proliferation following BrdU injection showed a similar frequency of BrdU^+^ ETP in WT and *Ltbr^−/−^* mice (Fig. 1C), arguing against the notion that the ETP reduction in *Ltbr^−/−^* mice was due to alterations in their ability to proliferate intrathymically. Despite these changes, total thymocyte numbers in WT and *Ltbr^−/−^* mice were comparable, including numbers of CD4^−^CD8^+^ immature single-positive and CD4^+^CD8^+^ cells (Fig. 1D), suggesting that increased thymocyte expansion during early stages of T cell development may restore normal thymocyte cellularity. Indeed, analysis of BrdU incorporation showed an increased frequency of BrdU^+^ DN3 thymocytes in *Ltbr^−/−^* mice compared with WT mice (Fig. 1E). Taken together, these findings show that LTβR controls the frequency of ETP in the adult, suggesting a role in the regulation of lymphoid progenitor entry to the thymus. They also indicate that ETP reduction in *Ltbr*^−/−^ mice is compensated by enhanced DN3 stage thymocyte proliferation that restores thymus cellularity to normal levels.

### Stromal cell expression of LTβR controls thymus entry of T cell progenitors

**FIGURE 2.**
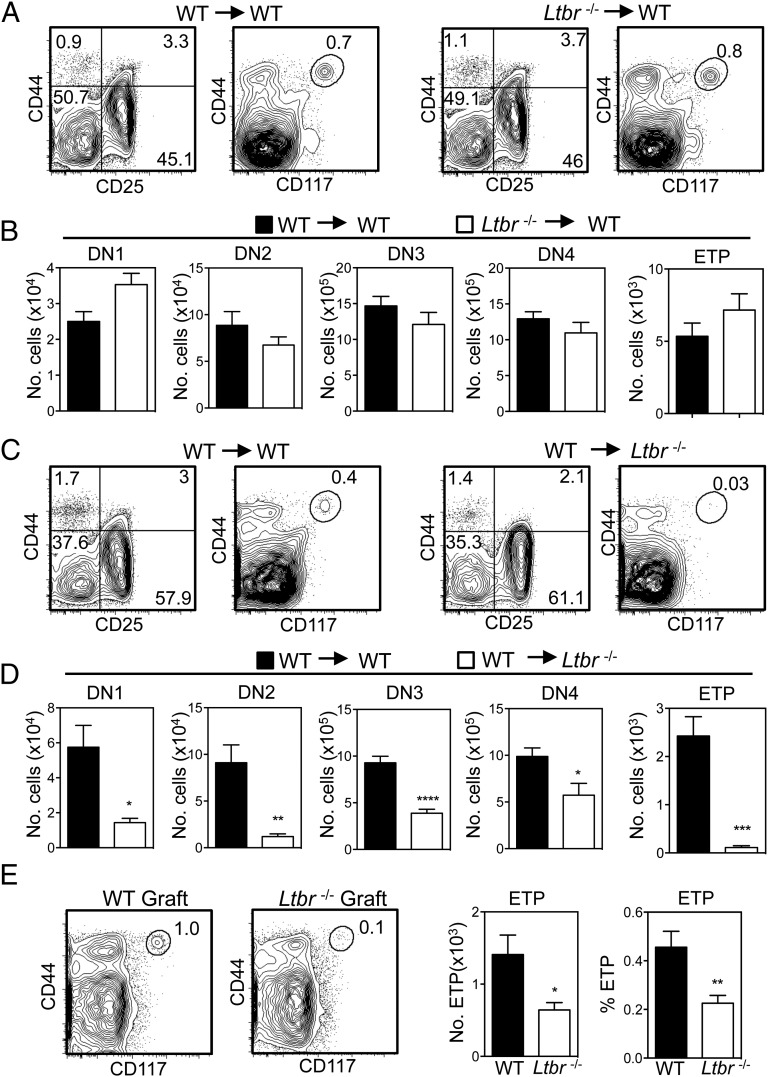
LTβR expression by thymic stroma controls thymus entry. (**A**–**D**) Lethally irradiated WT/*Ltbr^−/−^* mice were reconstituted with congenically marked T cell–depleted WT/*Ltbr^−/−^* BM cells as indicated. Representative FACS plots are shown, after gating on congenically marked donor-derived thymocytes (*n* = 10 from three independent experiments). (**E**) Frequency and absolute number of ETP in WT and *Ltbr^−/−^* dGuo thymus grafts following transplant into WT hosts for 6–8 wk (*n* ≥ 8 grafts from three independent experiments). Representative FACS plots are shown. *p < 0.05, **p < 0.01, ***p < 0.001, ****p < 0.0001.

Although LTβR regulates the development and function of thymic stromal microenvironments (25, 37), it can also directly influence hemopoietic cells (38). To investigate the LTβR-expressing cellular compartment that regulates progenitor entry to the thymus, we first established a series of reciprocal BM chimeras to confine LTβR expression to either stromal cells or hemopoietic cells. Initially, WT mice at 8 wk of age were lethally irradiated and injected i.v. with congenically marked T cell–depleted BM obtained from either WT or *Ltbr^−/−^* donor mice. Additionally, *Ltbr^−/−^* adult recipients were reconstituted with congenic T-depleted BM preparations from either WT or *Ltbr^−/−^* donors. Mice were harvested after 8 wk, and analysis of thymocyte development from donor-derived progenitors was performed. In chimeras using WT hosts, similar ETP proportions and numbers were generated from both WT and *Ltbr^−/−^* BM, and no significant alterations in early stage T cell development were observed (Fig. 2A, 2B). In contrast, a dramatic reduction in ETP frequency and proportion was observed when *Ltbr^−/−^* hosts were reconstituted with WT donor BM (Fig. 2C, 2D). Alterations in early thymocyte development, including a reduction in DN1–3 T cell progenitor compartments, were also noted in WT → *Ltbr^−/−^* chimeras (Fig. 2D). Next, to directly assess whether LTβR expression by thymic stroma is involved in progenitor entry to the thymus, we transplanted alymphoid WT or *Ltbr^−/−^* thymus lobes under the kidney capsule of WT mice. In this setting, LTβR is exclusively absent from stromal cells in the transplanted thymus. Importantly, flow cytometric analysis 6–8 wk after grafting showed a significant reduction in the proportion and absolute number of ETP in *Ltbr^−/−^* grafts compared with WT (Fig. 2E). Collectively, these results indicate that LTβR expression by thymic stroma is important for lymphoid progenitors to enter the thymus, a finding consistent with the cell-intrinsic requirement for LTβR in the development and function of thymic stromal microenvironments (25, 37, 39).

### LTβR differentially controls known regulators of thymus seeding

**FIGURE 3.**
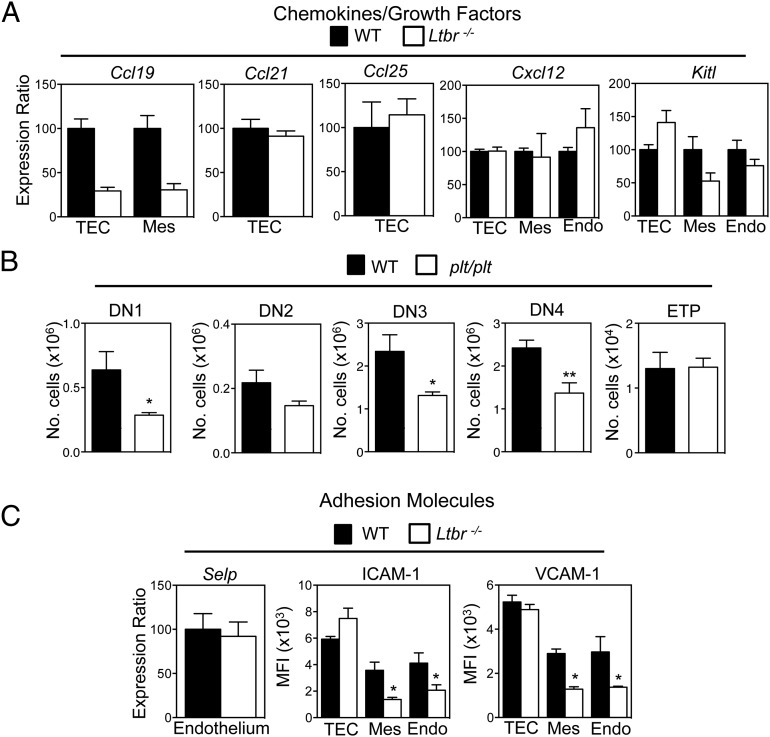
LTβR differentially regulates known mediators of thymus seeding. (**A**) Purified stromal samples from WT and *Ltbr*^−/−^ mice were analyzed by qPCR for the indicated genes. mRNA levels were normalized to β-actin (mean ± SEM) and represent at least two independent biological experiments. (**B**) Frequency of DN thymocyte subsets and ETP in thymuses from adult WT and *plt/plt* mice (*n* ≥ 9 from three independent experiments). (**C**) Comparison of *Selp* mRNA expression in WT and *Ltbr*^−/−^ endothelium, and mean fluorescence intensity (MFI) analysis of VCAM-1 and ICAM-1 in the indicated stromal subsets of WT and *Ltbr^−/−^* mice. *p < 0.05, **p < 0.01.

To investigate how LTβR influences T cell progenitor entry to the thymus, we compared the expression of known regulators of thymus seeding in purified thymic stromal subsets from WT and *Ltbr^−/−^* mice. *Ccl25* and *Cxcl12* mRNA levels were not altered in *Ltbr^−/−^* mice (Fig. 3A), suggesting that the requirement for LTβR is not explained by its regulation of ligand availability for the chemokine receptors CCR9 and CXCR4. Similarly, we saw no substantial alteration in *Kitl* expression, an important growth factor for immature T cell progenitors (40, 41). Interestingly, we also found no differences in *Ccl21* mRNA levels in WT and *Ltbr*^−/−^ TEC (Fig. 3A). Importantly, however, note that this observation is not incompatible with an earlier study showing that CCL21^+^ medullary TEC (mTEC) are present, but at a reduced frequency in *Ltbr*^−/−^ mice (25), and may simply reflect differences in methods (qPCR/flow cytometry) used to measure CCL21 expression. Interestingly, however, we did see decreased expression of *Ccl19* mRNA in both TEC and thymic mesenchyme from *Ltbr^−/−^* mice (Fig. 3A), suggesting that LTβR could play a role in the recruitment of T cell progenitors via regulation of intrathymic CCR7 ligand availability. To investigate this directly, we examined ETP frequency and early T cell development in *plt/plt* mice that lack expression of CCL19 and CCL21 (32). Interestingly, whereas our analysis of *plt/plt* mice showed alterations in DN1–4 T cell progenitor frequencies that are consistent with an earlier report (24), we saw no differences in ETP numbers in WT and *plt/plt* thymuses (Fig. 3B). This agrees with other studies showing that the major impact of CCR7 deficiency on ETP requires the combined absence of CCR9 (22, 23, 27). Thus, mice lacking either LTβR or CCR7 ligands do not share alterations in ETP frequency, suggesting that control of CCL19 and CCL21 expression by LTβR does not explain its requirement during thymus seeding.

In addition to chemokines, thymus entry requires attachment of lymphoid progenitors to stromal cells expressing the adhesion molecules P-selectin, VCAM-1, and ICAM-1 (17, 19, 20). We found that *Selp* mRNA levels were comparable in WT and *Ltbr^−/−^* thymic endothelium (Fig. 3C). In contrast, levels of VCAM-1 and ICAM-1 were altered. Specifically, both *Ltbr^−/−^* thymic endothelium and mesenchyme had significantly reduced levels of VCAM-1/ICAM-1 (Fig. 3C). Interestingly, levels of expression on WT and *Ltbr^−/−^* TEC were comparable (Fig. 3C). Collectively, our findings demonstrate a differential requirement for LTβR in the control of adhesion molecule expression by TEC and non-TEC stroma, and they show that within the latter, adhesion molecules known to influence thymus entry can be subdivided into LTβR-dependent (VCAM-1/ICAM-1) and LTβR-independent (P-selectin) groups.

### LTβR mediates thymus recovery after BMT

**FIGURE 4.**
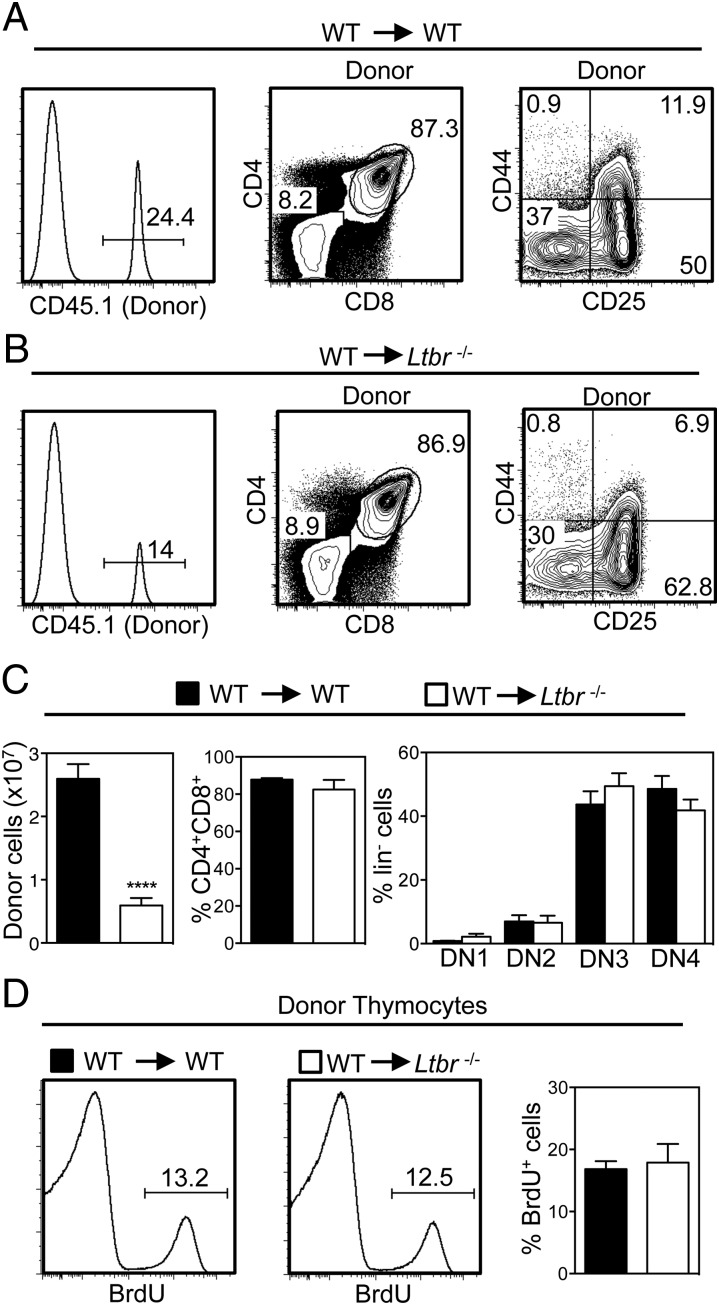
Initial thymic reconstitution after BMT is controlled by LTβR. Lethally irradiated WT (**A**) and *Ltbr*^−/−^ (**B**) mice were reconstituted with T-depleted congenically marked WT BM cells and harvested after 13 d. Thymic reconstitution was determined by calculating the intrathymic frequency of CD45.1^+^ donor cells, and bar charts in (**C**) show numbers of total donor thymocytes and percentages of donor-derived double-positive and DN thymocytes. *n* ≥ 13 from five independent experiments; representative FACS plots are shown. (**D**) Analysis of BrdU incorporation in WT donor-derived CD45.1^+^ thymocytes from WT and *Ltbr^−/−^* hosts (*n* = 6 from three independent experiments). ****p < 0.0001.

T cell progenitor recruitment to the thymus influences T cell reconstitution and thymus recovery following ablative therapy and BMT (29). We next examined the possible role of LTβR in this context and focused on early events in thymus reconstitution (42–44). Importantly, other studies have shown that donor-derived ETP are not detectable in irradiated mice at time points shortly after BMT, with a clearly defined ETP population not apparent until after 3 wk following transplant (45). Thus, to assess the role of LTβR in early phases of thymus recovery, we determined the frequency and number of donor-derived congenically marked thymocytes 13 d after the transplantation of WT BM into lethally irradiated WT and *Ltbr^−/−^* mice. Importantly, at early stages of thymus recovery, note that the thymus is dominated by thymocytes of host origin that survive and expand after irradiation (Fig. 4A, 4B) (43, 44). Furthermore, although 2–3 × 10^7^ CD45.1^+^ donor-derived thymocytes were recovered from the thymuses of irradiated WT hosts, a frequency that is in line with other studies (46), we saw a significant 3- to 4-fold reduction in the number of CD45.1^+^ donor-derived thymocytes recovered from *Ltbr^−/−^* hosts (Fig. 4A–C). Interestingly, the pattern of development (Fig. 4A–C) and frequency of BrdU^+^ cells (Fig. 4D) in donor-derived thymocytes was comparable in WT and *Ltbr^−/−^* hosts, indicating that this difference was not due to an inability of donor progenitors to undergo proliferation and differentiation in the irradiated *Ltbr^−/−^* host thymus. Rather, the reduced frequency of donor-derived thymocytes in the thymus of *Ltbr^−/−^* hosts suggests that as in the steady-state, progenitor entry to the thymus after BMT involves LTβR.

### In vivo agonistic anti-LTβR treatment enhances thymus recovery after BMT

**FIGURE 5.**
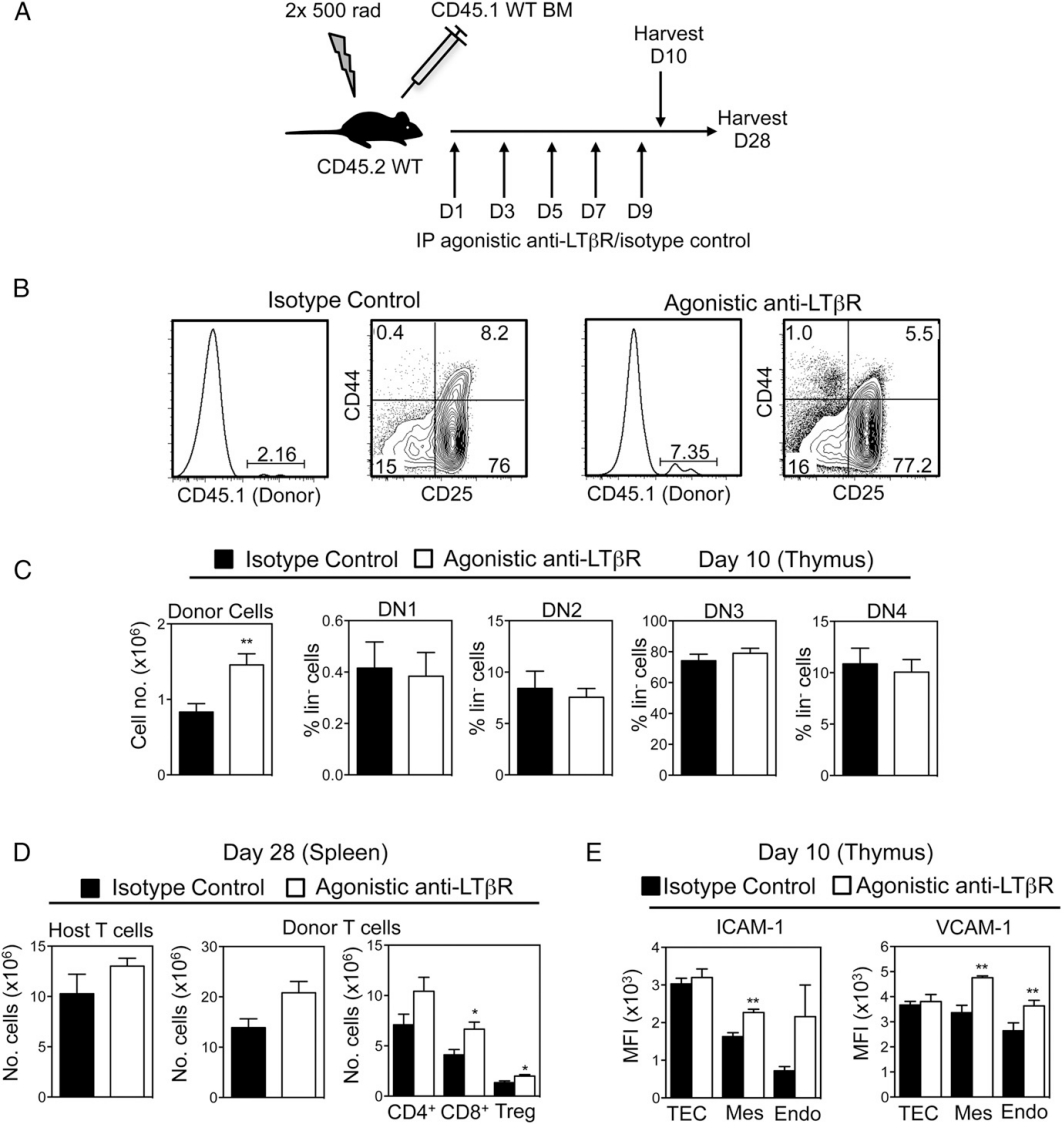
LTβR stimulation enhances thymic reconstitution after BMT. (**A**) Lethally irradiated WT mice were reconstituted with T-depleted congenic WT BM cells, injected i.p. with 100 μg of agonistic anti-LTβR or isotype on days 1, 3, 5, 7, and 9, and harvested on day 10 or 28. (**B** and **C**) Thymic reconstitution was determined by calculating the intrathymic frequency of total CD45.1^+^ donor thymocytes and donor DN thymocyte subsets at day 10. Representative FACS plots are shown (*n* ≥ 8 from three independent experiments). (**D**) Frequencies of host- or donor-derived splenic T cells were determined at day 28. (**E**) Mean fluorescence intensity (MFI) expression of VCAM-1 and ICAM-1 on CD45^−^EpCAM1^+^ TEC, CD31^+^podoplanin^−^ endothelium, and CD31^−^podoplanin^+^ mesenchyme from mice treated with either anti-LTβR or control Ab control treated mice at day 10. *n* ≥ 8 from two independent experiments. *p < 0.05, **p < 0.001.

Given our findings on thymus reconstitution in *Ltbr^−/−^* mice, we next investigated whether exogenous LTβR stimulation may be a potential therapeutic means to boost thymus recovery after BMT. WT mice were lethally irradiated and then reconstituted with congenic CD45.1^+^ T cell–depleted BM preparations. The day after BMT, mice then received either 100 μg of agonistic anti-LTβR (35) or an isotype control Ab, every other day until day 9 (Fig. 5A). Tissues were harvested from chimeric mice on days 10 and 28 after BMT, and donor-derived thymocytes and T cells were analyzed by flow cytometry. Again, at this early day 10 time point (Fig. 5B), the dominant population of cells in the thymus were host-derived thymocytes that survived irradiation. Importantly, however, although ETP cannot be detected at this early time point (45), we found that a population of donor-derived thymocytes was detectable, representing initial donor engraftment of the host thymus (Fig. 5B). Strikingly, 10 d after transplant, the proportion and absolute number of donor-derived CD45.1^+^ thymocytes was significantly increased in mice receiving agonistic LTβR compared with isotype control (Fig. 5B, 5C), and cells showed a normal pattern of progression through DN thymocyte stages at this early posttransplant time point (Fig. 5B, 5C). Furthermore, analysis of chimeras 28 d after transplant showed a significant increase in donor-derived peripheral T cell numbers in the spleens of mice receiving agonistic anti-LTβR (Fig. 5D). Interestingly, this effect of anti-LTβR was specific, as residual host-derived splenic T cell numbers were not affected by Ab treatment (Fig. 5D). Thus, our data suggest that agonistic anti-LTβR treatment enhances the recovery of thymopoiesis by increasing the frequency of donor-derived progenitors in the thymus of irradiated recipient mice, and this leads to an increase in donor-derived T cells in the periphery.

Finally, given our data suggesting that LTβR may influence thymus seeding by regulating expression of VCAM-1 and ICAM-1, we analyzed the impact of anti-LTβR treatment on their levels in thymic stroma after BMT. Lethally irradiated mice were reconstituted with T cell–depleted BM and subjected to anti-LTβR/isotype control treatment, and thymuses were harvested after 10 d. Following digestion, CD45^−^EpCAM1^+^ TEC, CD45^−^CD31^+^ endothelium, and CD45^−^podoplanin^+^ mesenchymal stromal cells were analyzed by flow cytometry. Interestingly, in mice receiving anti-LTβR treatment, both thymic endothelium and mesenchymal cells showed increased levels of both VCAM-1 and ICAM-1 whereas levels on TEC were not altered (Fig. 5E). Collectively, these observations show that in vivo stimulation of LTβR boosts thymic recovery after BMT, and this correlates with enhanced expression of adhesion molecules known to facilitate thymic entry of T cell progenitors in non-TEC stroma.

## Discussion

The absence of an intrathymic hemopoietic stem cell pool means that to sustain T cell production throughout life, the thymus must continuously import lymphoid progenitors from extrathymic sites. Additionally, the entry of donor-derived lymphoid progenitors to the thymus represents a rate-limiting step in re-establishing T cell–mediated immunity that follows ablative therapy and BMT. Despite this importance, relatively few regulators of T cell progenitor entry to the thymus are known. Collectively, the work described in the present study shows that the TNFRSF member LTβR plays a key role in the regulation of thymus seeding. We also show LTβR is involved in thymus entry in both the steady-state and during the early phases of thymus recovery that take place after BMT. Regarding the latter, manipulation of the LTβR axis using agonistic Abs significantly improved donor-derived thymopoiesis and boosted T cell recovery after BMT, suggesting that LTβR stimulation is part of a common mechanism that controls thymus entry in both the steady-state and during immune reconstitution.

Our finding that the requirement for LTβR maps to thymic stromal cells is significant, as it extends our understanding of its importance in the regulation of thymus function. For example, identification of a role for LTβR in thymic entry complements work demonstrating its importance for the thymic egress of mature thymocytes (37). Interestingly, another study has shown that mature thymocytes and T cell progenitors are present within the same perivascular spaces that surround intrathymic blood vessels (47). Taken together, these findings raise the possibility that the limited entry of T cell progenitors to the *Ltbr^−/−^* thymus is caused by an accumulation of mature thymocytes at a common site of thymic exit and entry. Further experiments are required to examine the possible relationship between these processes and the mechanisms that regulate them.

Although LTβR has been shown to influence the frequency of CCL21^+^ mTEC (25), our findings, including normal ETP frequency in *plt/plt* mice, suggest that its involvement in thymus seeding is not simply explained by its regulation of CCR7 ligands. However, whether the requirement for LTβR shown in this study is linked to the noted decrease in availability of CCL21-expressing mTEC^lo^ cells (25) is currently not clear. Importantly, the positive impact of LTβR stimulation on thymic reconstitution shown in this study is observed shortly after BMT, a time point at which thymus seeding is transiently independent of CCR7 and CCR9 (29), further suggesting that LTβR stimulation augments thymic reconstitution via mechanisms other than chemokine availability. As indicated from studies on peripheral lymphoid tissues (48), another way in which LTβR may influence thymus entry is through its ability to regulate the expression of adhesion molecules by endothelium and/or mesenchyme. This would fit well with our demonstration of reduced levels of VCAM-1/ICAM-1 in steady-state *Ltbr^−/−^* mice, and their enhanced expression in both thymic endothelium and mesenchyme following in vivo anti-LTβR treatment. It is also supported by previous studies in which Ab-mediated blockade of VCAM-1/ICAM-1 inhibited thymic entry of transferred lymphoid progenitors (20). Interestingly, unlike VCAM-1 and ICAM-1, we found that LTβR did not control P-selectin expression, a finding that highlights its differential ability to influence expression of adhesion molecules linked to thymus entry. Taken together, these data raise the possibility that LTβR regulates thymic entry through control of integrin-mediated firm adhesion and transendothelial migration, rather than initial phases of selectin-mediated rolling and chemokine-driven activation/migration. Alternatively, the requirement for LTβR may relate to its importance in the regulation of medullary microenvironments. Thus, altered mTEC development and organization in *Ltbr^−/−^* mice (25, 37) may limit the ability of the thymus to attract migrant lymphoid progenitors. However, it is interesting that whereas thymus medulla disorganization is also evident in *plt/plt* mice (24), we found that their ETP frequency was not altered.

Finally, the identity of the LTβR ligands and the cells they are expressed by that influence thymus seeding are not known. Relevant to this, earlier studies have shown the difficulty in correlating known roles for LTβR in thymus development and function with the availability of the LTβR ligands lymphotoxin and LIGHT. For example, whether defects in thymus organization caused by absence of LTβR are mirrored in mice lacking LTβR ligands, either individually or in combination, is not certain (37, 49). However, as thymic expression of LTβR ligands has been mapped to a variety of hemopoeitic cells (37, 50), we suggest that the requirement for LTβR during thymus entry represents a further example of how thymic crosstalk regulates the TNFRSF-mediated control of thymus function. In summary, our study identifies a new role for LTβR in the control of thymus function, where it acts as a regulator of the earliest phases of T cell development by influencing the intrathymic availability of the earliest thymocyte progenitors. That this role extends to thymic recovery after BMT suggests the potential of LTβR as an immunotherapeutic target to boost T cell recovery and restore essential immune system functioning following ablative therapy.
